# Molecular targeted therapy in advanced renal cell carcinoma: A review of its recent past and a glimpse into the near future

**DOI:** 10.4103/0970-1591.57899

**Published:** 2009

**Authors:** John S. P. Yuen

**Affiliations:** Department of Urology, Singapore General Hospital, Outram Rd, Singapore 169 608, Republic of China

**Keywords:** Molecular targeted therapy, renal cell carcinoma

## Abstract

Renal cell carcinoma (RCC) is the most lethal of all urologic malignancies. Recent translational research in RCC has led to the discovery of a new class of therapeutics that specifically target important signaling molecules critical in the pathogenesis of the disease. It is now clear that these new molecular targeted agents have revolutionized the management of patients with metastatic RCC. However, the exact molecular mechanism accounting for their clinical effect is largely unknown and a significant proportion of patients with metastatic RCC do not respond to these therapeutics. This review presents the relevant background leading to the development of molecular targeted therapy for patients with advanced RCC and summarizes current management issues in particular relating to the emerging problem of treatment resistance and the need for clinical and laboratory biomarkers to predict treatment outcomes in these patients. In addition, this paper will also address surgical issues in the era of molecular targeted therapy including the role of cytoreductive surgery and surgical safety issues post-molecular therapy. Lastly, this review will also address the need to explore new molecular treatment targets in RCC and briefly present our work on one of the promising molecular targets - the type 1 insulin-like growth factor receptor (IGF1R), which may in the near future lead to the development of anti-IGF1R therapy for patients with advanced RCC.

## INTRODUCTION

Surgery by radical nephrectomy remains the mainstay of curative treatment for patients who present with early-stage RCC. However, a significant proportion of patients develop metastatic disease after RCC surgery and the incidence depends on tumor stage and grade: occurring in 0–7% and 5–26% of patients with pT1 or pT2 tumors, respectively and 9% or 61% of patients with Grade 1 or Grade 2 tumors, respectively.[1–7] For those who present late with advanced and metastatic disease, the overall clinical course of RCC varies; approximately 50% of patients survive less than 1 year and 10% survive for more than 5 years.[8] Chemotherapy has consistently been shown to be an ineffective form of treatment for this disease.[9,10] In fact, RCC is one of the most chemo- and radio-resistant of all human solid tumors. Until recently, the only effective treatment for metastatic disease was cytokine-based immunotherapy with interferon (IFN)-α or interleukin (IL)-2, which produce a response rate of only 10-15%.[11,12] However, recent advances in the understanding of biology and genetics of RCC have led to the emergence of novel molecular targeted approaches for the treatment of metastatic RCC (mRCC).

**Figure 1:**
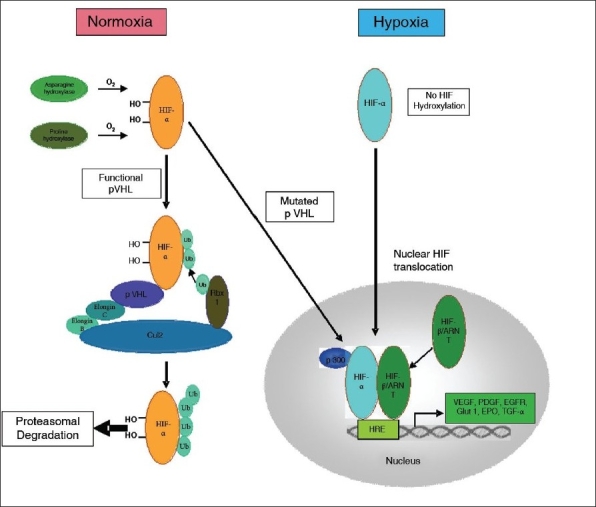
pVHL/HIF oxygen sensing pathway. In normoxia, HIF-α is hydroxylated at two proline residues and an asparagine residue via oxygen-dependent enzymatic mechanisms. Asparagine hydroxylation blocks HIF-α interaction with transcriptional coactivator p300. Proline hydroxylation allows binding of HIF-α to wild-type pVHL, which promotes ubiquitination and proteasomal degradation of HIF-α. In hypoxia, or in the absence of functional pVHL, HIF-α is not degraded, but translocates to the nucleus forming a heterodimer with HIF-β/ARNT. The HIF-α/β heterodimer activates transcription at hypoxia-responsive elements (HRE), resulting in expression of hypoxia-inducible genes such as vascular endothelial growth factor (VEGF), platelet-derived growth factor (PDGF), epidermal growth factor receptor (EGFR), glucose transporters (e.g. GLUT-1), erythropoietin (EPO) and transforming growth factor-α (TGF-α)

**Figure 2:**
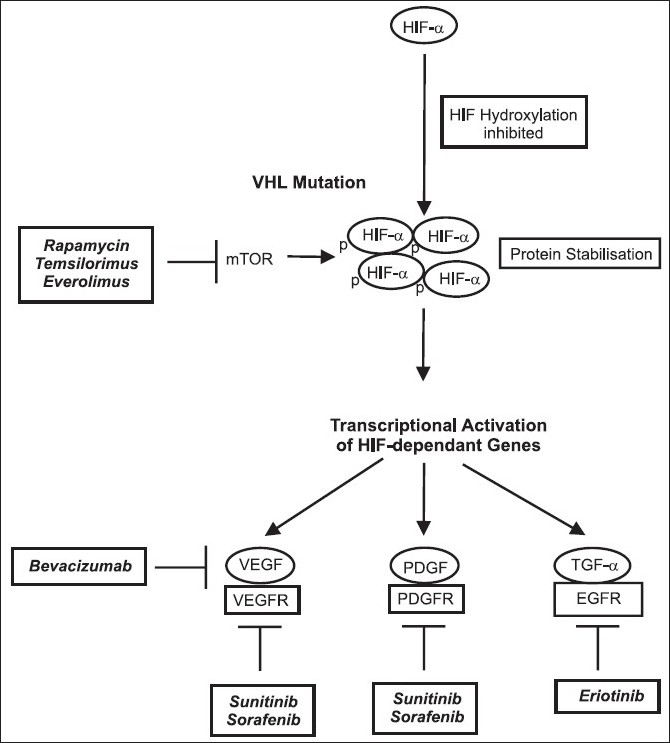
HIF stabilisation secondary to VHL mutations and downstream activation of HIF-dependant gene products as molecular targets for patients with metastatic CC-RCC

As all hereditary VHL-related and up to ^~^75% of sporadic clear cell RCC (CC-RCC) harbour biallelic von *Hippel-Lindau (VHL)* gene inactivation, this leads to constitutive activation of hypoxia signaling in tumor cells [Figure 1] with resultant upregulation of angiogenic factors including the vascular endothelial growth factor (VEGF) and platelet derived growth factor.[13,14] These angiogenic factors, which cause tumors to become highly vascular and thus play a critical role in CC-RCC growth and biology[15] have emerged as treatment targets in patients with mRCC [Figure 2]. The following sections describe the discovery, structure, and function of VHL in the context of its role in the pathogenesis of CC-RCC.

## VHL GENE

The VHL tumor suppressor gene was cloned in 1993. It is located on chromosome 3p25-26 and consists of 3 exons that encode a 213 amino-acid protein (pVHL) with a molecular weight of ˜24 to 30 kDa (VHL_30_).[16] A second pVHL isoform of 160 amino acids of approximately 19 kDa (VHL_19_) is produced as a result of internal translational initiation at an in-frame start codon (ATG) at codon 54.[17–20] Both isoforms apprear to retain tumor suppressor activity perhaps accounting for the paucity of pathogenic mutations affecting the first 50 amino acid residues. For simplicity, in this review pVHL is used when referring to both isoforms generically.

## PVHL STRUCTURE AND FUNCTIONAL DOMAINS

**Figure 3:**
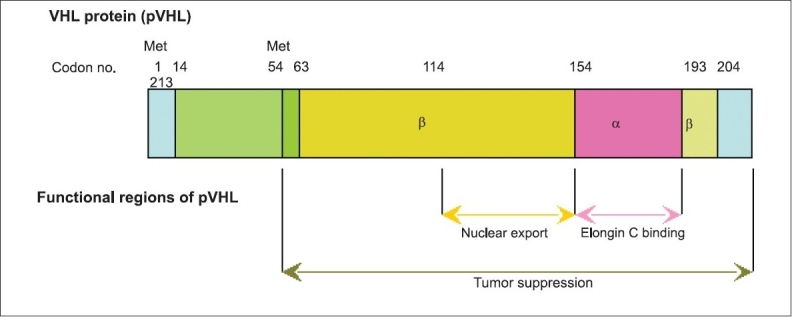
von Hippel-Lindau (VHL) protein structure and function. The (- and (-domain structure of the VHL protein (codon numbers 1-213), and the two methionine (Met) start condons (at codons 1 and 54), are shown. Functional regions of VHL protein (pVHL) are indicated

pVHL has two major structural domains [Figure 3]. The β-domain consists of a seven-stranded β sandwich and one α-helix spanning amino acids 63–154 and 193–204, respectively. The smaller, α-domain (amino acids 155–192) consists of three α-helices.[21] The α-domain recruits the elonginC/elonginB/CUL2/Rbx1 complex [Figure 1] and the β-domain interacts with the hydroxylated oxygen-dependent degradation (ODD) domain of hypoxia-inducible factor (HIF)-α subunits.[22–24] The α-domain is a hot spot for missense mutations in VHL (e.g., Arg167) and these often mutated amino acids have been identified as being involved in direct interaction with the elonginC/elonginB/CUL2/Rbx1 complex or in interactions with other residues to stabilise the structure of the α-domain (Kaelin and Maher, 1998). Missense mutations are also frequently observed in codons 8-122 encoding an area on the surface of the β-domain opposite the binding site for elongin C involved in binding with the ODD of HIF-α subunits.[25]

## FUNCTIONS OF PVHL - THE VHL/HIF OXYGEN SENSING PATHWAY

The role of VHL as a tumor suppressor is principally mediated via its interaction with HIFs. In addition, some VHL mutations fall outside the region involved in the regulation of HIFs, suggesting that pVHL possesses HIF-independent functions.[26–28] One of the most well-studied functions of pVHL relates to its ability to regulate protein expression at the post-transcriptional level. pVHL forms a stable complex that contains elongin B, elongin C, cullin 2 (Cul2), and Rbx1[29,30] and acts as an E3 ubiquitin ligase for ubiquitination and proteasomal degradation of targeted proteins. pVHL acts as the substrate recognition and binding subunit of this complex. Following substrate binding, these complexes are capable of directing the covalent attachment of polyubiquitin tails to the bound proteins, serving as signals for such ubiquinated protein to be degraded by the proteasome.[31] One of the most important pVHL-regulated proteins is HIF-1, a heterodimeric sequence-specific, DNA-binding transcription factor composed of HIF-1α and HIF-1β (also known as the aryl hydrocarbon receptor nuclear translocator ARNT) subunits.[32] The HIF-1β subunit is expressed constitutively, and the biological activity of HIF-1 is regulated by the expression of and activity of HIF-1α.[33] There is a family of HIF-α proteins, including HIF-1α, HIF-2α, and HIF-3α, which is among the best-understood pVHL target proteins. The HIF-α subunits are highly unstable except under hypoxic conditions. In the presence of oxygen, HIF-α subunits are hydroxylated on conserved prolyl residues within the ODD by members of the elg-nine (EGLN) family[34–36] and on a specific asparagine residue by factor inhibiting HIF1 (FIH-1). The former reaction creates a binding site for pVHL at its β domain and the latter prevents transcription coactivator recruitment. pVHL-bound HIF- undergoes polyubiquitination and is subsequently degraded by the proteasome[36–38] [Figure 1]. Tumor-associated missense mutations in the β-domain of pVHL abrogate binding and degradation of HIF-α. Similarly, HIF-α is stabilised by mutations in the α-domain of pVHL that prevent the formation of the pVHL ubiquitin ligase complex.[24,36,39,40] In cells that lack functional pVHL, or in hypoxia, HIF-as are not degraded leading to constitutive expression of HIF-α subunits that translocate into the nucleus and form heterodimers with HIF-β/ARNT, activating the transcription of a range of genes involved in cellular adaptation to hypoxia. These hypoxia-inducible genes include genes that regulate angiogenesis (VEGF and PDGF), glucose uptake and metabolism (Glut 1 glucose transporter), extracellular pH (carbonic anhydrase IX), erythopoiesis (erythropoietin), and mitogenesis (including transforming growth factor-α, TGF-α, and platelet-derived growth factor-B [PDGF-B]).[41,42]

## MOLECULAR TARGETED THERAPY FOR RCC

The discovery of the molecular links underlying the relationship between VHL, hypoxia signalling, and VEGF in the biology of CC-RCC has identified a pathway that is a potential treatment target. Many novel molecular targeted therapeutic agents, including small molecule tyrosine kinase inhibitors (TKIs) and human monoclonal antibodies are currently undergoing pre-clinical and clinical trials. Of these, the small molecule inhibitors sunitinib and sorafenib inhibit activation of the tyrosine kinase domain of receptor tyrosine kinases (RTKs) including the vascular endothelial growth factor receptor (VEGFR) and platelet-derived growth factor receptor (PDGFR) and temsirolimus that targets the mammalian target of rapamycin (mTOR) pathway. These small molecule inhibitors, together with bevacizumab, a monoclonal VEGF antibody, have shown antitumor activity in randomised clinical trials.[43–46] All three small molecule inhibitors, sunitinib, sorafenib, and temsirolimus have been approved by the U.S. Food and Drug Administration (FDA) for treatment the of mRCC. These molecular targeted therapeutics have effectively changed patient management of mRCC. As discussed in the following sections, all three have been shown to be more effective than cytokine-base therapies, which are relatively ineffective.[11,12] A description of the clinical development of these and other novel therapeutic agents is summarised in Table 1.

**Table 1:** Selected targeted agents for metastatic renal cell carcinoma

Agent	Class	Mechanism of action	Clinical trial Phase	FDA approved for RCC
Sorafenib	Small-molecule	TKI of VEGFR, PDGFR, Ras	II, III	√Dec 2005
Sunitinib	Small-molecule	TKI of VEGFR, PDGFR	II, III	√Jan 2006
AG-0736	Small-molecule	TKI of VEGFR, PDGFR	II	
Pazopanib	Small-molecule	TKI of VEGFR, PDGFR	II, III	
PTK787	Small-molecule	TKI of VEGFR, PDGFR	I	
zImatinib	Small-molecule	TKI of PDGFR	II	
Gefitinib	Small-molecule	TKI of EGFR	II	
Erlotinib	Small-molecule	TKI of EGFR	II	
Lapatinib	Small-molecule	TKI of EGFR/Erb2	II, III	
Temsirolimus	Small-molecule	mTOR inhibitor	II, III	√ May 2007
RAD001	Small-molecule	mTOR inhibitor	II	
Bortezomib	Small-molecule	Inhibitor to 26s proteosome	II	
Cetuximab	Monoclonal antibody	Antibody to EGFR	II	
ABX-EGF	Monoclonal antibody	Antibody to EGFR	II	
Bevacizumab	Monoclonal antibody	Antibody to VEGF	II, III	
VEGF-Trap	Monoclonal antibody	Antibody to VEGF	I, II	
G250	Monoclonal antibody	Antibody to CA IX	II	

TKI, tyrosine kinase inhibitor; VEGF, vascular endothelial growth factor; VEGFR, vascular endothelial growth factor receptor; PDGFR, platelet-derived growth factor receptor; EFGR, epidermal growth factor receptor; CA IX, carbonic anhydrase IX; mTOR, mammalian target of rapamycin

## SUNITINIB

Sunitinib is an orally bioavailable small molecule inhibitor of multiple RTK kinase domains including VEGFR-1 and -2, PDGFR-α and -β, KIT receptor, and fms-related tyrosine kinase 3 (FLT3) receptor.[47,48] Two single-arm Phase II studies in patients with cytokine-refractory mRCC have shown partial response (PR) rates of 40–44% with an additional 22–27% of patients in stable disease (SD) for at least 3 months.[49,50] Based on these promising data, a randomised Phase III trial was conducted to compare the effects of sunitinib with IFNα in the first-line treatment of clear cell mRCC (CC-mRCC). This study has shown a median progression-free survival (PFS) of 11 months for patients taking sunitinib versus 5 months for IFNα (hazard ratio 0.42; *p* < 0.001). The response rate was 31% for sunitinib versus 6% for IFNα (*p* < 0.001).[45] These data demonstrate a significant improvement in PFS and objective response rate (ORR) for sunitinib over IFNα in the first-line treatment of CC-mRCC leading to the recommendation that sunitinib be considered as standard first-line treatment for this disease.[51] The recommended clinical regimen for sunitinib is 50 mg per day for a cycle of 4 weeks on followed by 2 weeks off (4/2 schedule).

## SORAFENIB (BAY 43-9006)

Sorafenib is an orally bioactive small molecule in the class of bis-aryl ureas that was initially found to inhibit the serine/threonine Raf-1 kinase. It has subsequently been found to inhibit several RTKs including VEGFR-2 and -3, PDGFR-β, FLT3 receptor, and c-KIT receptor.[47,52] A recently completed Phase II randomised-discontinuation study involving 202 patients with CC-mRCC has demonstrated that in patients in the SD arm who were randomised to receive sorafenib, the median PFS was 24 weeks after randomisation compared with 6 weeks in the placebo group.[53] This study demonstrated significant disease-stabilising activity in CC-mRCC and tolerability of treatment. A randomised Phase III trial comparing sorafenib with placebo showed the median PFS was 5.5 months in the sorfenib arm compared with 2.8 months in the placebo group (hazard ratio 0.44; *p* < 0.01).[43] This study also suggests an overall survival benefit in the sorafenib arm compared with placebo. These data demonstrate the clinical activity of sorafenib in CC-mRCC and led to the regulatory approval of the drug by the FDA as new treatment for patients with advanced RCC.

## TEMSIROLIMUS (CCI-779)

Temsirolimus is a derivative of the immunosuppressant rapamycin that forms a complex with FK-506 binding protein-12 to inhibit the activity of mTOR.[54] mTOR is a serine/threonine kinase that is activated following activation of RTKs upon binding of growth factors including VEGF, PDGF, and insulin-like growth factors (IGFs). The principal downstream effect of mTOR is the activation of translation initiation, resulting in increased translation of proteins including HIFs.[55] Given the critical role of HIFs in the oncogenesis of RCC, mTOR inhibition is an obvious therapeutic target for this disease.[56] A Phase II study involving 111 patients with refractory mRCC showed PR in 75% of the patients and clinical benefit (either complete response [CR], PR, or SD) for at least 24 weeks in approximately 50% of the patients.[51] A Phase III randomised trial with temsirolimus as a single agent versus temsirolimus plus IFNα versus IFNα alone as first-line treatment in poor-risk mRCC patients showed that temsirolimus significantly increases the survival of this group of patients compared with IFNα alone, with median survivals of 10.9, 7.3, and 8.4 months for temsirolimus, IFNα, and combination treatment, respectively.[44] Presumably, the greater effects of temsirolimus seen in poor-risk patients might be due to a higher incidence of phosphatase and tensin homologue (PTEN) activating mutations resulting in constitutive AKT activation in this cohort of patients.[57] Further studies are needed to assess the role of temsirolimus as first-line therapy for patients with a more favourable prognosis or as combined treatment with other agents.

## OTHER MOLECULAR THERAPIES

RCC is a highly vascular tumor associated with high expression of VEGFR and epidermal growth factor receptor (EGFR).[13,58] However, to date the results of single-agent clinical trials using monoclonal antibodies that block VEGFR (bevacizumab) or small molecule TKIs targeting EGFR (erlotinib) have been disappointing.[46,59,60] A Phase II trial in cytokine-refractory patients comparing placebo with 2 dose regimens of bevacizumab showed a modest response rate of 10% with the higher dose regimen.[46] Subsequently a reported Phase II trial with bevacizumab and erlotinib showed that 3% of the patients achieved CR, 22% of the patients achieved PR, and 61% of the patients achieved SD following 8 weeks of treatment with a median time to progression of 11 months.[61] A Phase II trial evaluating bevacizumab alone versus bevacizumab and erlotinib has shown that the addition of erlotinib to bevacizumab did not result in additional clinical benefit compared with bevacizumab alone in first-line treatment of mRCC.[62] It is unclear if RCC patients who respond to the combination of bevacizumab and erlotinib possess similar gain-of-function mutations within the EGFR tyrosine kinase domain as has been documented in erlotinib-sensitive patients with non-small cell lung cancer.[63,64]

### After the initial excitement, what is next? Limitations of the current molecular targeted therapy in RCC and scopes for further research

The two small molecule TKIs currently FDA-approved for mRCC (sunitinib and sorafenib) are however not TKR-specific. Despite the many trials reporting clinical efficacies of this new class of therapeutics, the exact molecular mechanism(s) accounting for their clinical effects is still largely unknown. While the clinical efficacy of molecular targeted therapy in patients with mRCC is impressive in some patients, approximately 60% of patients with mRCC do not response to these TKIs. It is now obvious that there are inherent limitations and disadvantages with the use of these therapeutics as monotherapy agents. It is hypothesised that monotherapy with any single TKI can potentially be limited by tumor cell adaptation and compensation with overexpression of non-targeted oncogenic growth factor or TKRs that confer resistance to the tumor cells. This hypothesis is supported by the observation that for those patients who showed response, the duration of clinical response was typically about 10 to 12 months[45] during which clonal expansion of resistant tumor cells may occur.

## TREATMENT RESISTANCE AND WHAT CAN BE OFFERED

The major challenges facing clinicians treating patients with advanced RCC are the lack of clinical and laboratory parameters to predict treatment response; and for those who responded to these new therapeutic agents, the emergence of patients who developed resistance to the therapy. Indeed, there has been a flurry of activities directed to develop a second-line treatment strategy for the increasing number of patients who had shown initial response but later developed resistance to the molecular agents. One strategy is to employ sequential or combination targeted therapy. One example is that for patients with disease progressing under sunitinib, the administration of sorafenib still yields an objective response rate of 18%.[65] The other approach is to use other class of targeted therapy either in combination or in sequential therapy after the development of treatment resistance. It has recently been reported that an oral mTOR inhibitor, everolimus, was shown in a Phase III randomised controlled trial to result in prolongation of disease PFS in patients with mRCC who had progressed on VEGF-targeted therapy.[66] The current recommendations for the first-line and second-line molecular targeted therapy for patients with mRCC is summarised in Table 2. Current evidence seems to suggest that there is no cross resistance among the molecular targeted therapeutic agents; however, there are currently no identifiable factors to predict treatment response following first-line treatment with the suggested second-line agents. In the near future, it is anticipated that new and more efficacious targeted agents will be developed to augment the clinical effect of currently available agents as first- or second-line therapy for these patients. One of the promising new molecular targets is the IGF1R as described in the following section. As IGF1R signalling is upstream of the molecular targets inhibited by the currently used small molecule inhibitors, it remains to be seen if a combination of IGF1R inhibition and the currently available small molecule inhibitors will result in better efficacy and more prolonged clinical effects.

**Table 2:** mRCC Treatment Algorithm

	Setting	Therapy (Level 1)
Treatment naïve patient	MSK Risk: Good or intermediate	Sunitinib Bevacizumab + IFα
	MSK Risk: Poor	Temsirolimus
Treatment	Cytokine Refractory	Sorafenib
Refractory patient	Refractory to VEGF/VEGFR Inhibitors	Everolimus
	Refractory to mTOR Inhibitors	Investigational

## UNRESOLVED ISSUES OF MOLECULAR TARGETED THERAPY AND SURGERY

### Cytoreductive surgery in the era of molecular targeted therapy

There is currently no available level-one data recommending cytoreductive surgery before commencement of systemic molecular targeted therapy. Proponents for cytoreductive surgery would cite the established practice and benefit of pre-immunotherapy cytoreductive nephrecotmy. The results of the South-West Oncology Group (SWOG) trial 8949[67] and the European Organisation for Research and Treatment of Cancer (EORTC) trial 30947,[68] demonstrated survival benefit for patients who underwent cytoreductive surgery before systemic IF-α when compared with patients treated with immunotherapy alone. A subsequent pool analysis of these two trials demonstrated a superior survival benefit with median survival of 13.6 months for patients who underwent cytoreductive nephrectomy and IF-α compared with 7.8 months for patients treated with IF-α alone.[69]

While data from the immunotherapy trials may not have any bearing on the role of cytoreductive surgery in the era of molecular targeted therapy, one can derive some conclusions from the fact that the therapeutic efficacy of molecular targeted therapy have largely been observed in patients who had prior cytoreductive surgery as the overwhelming majority of patients in the Phase II/III studies of targeted molecular therapy underwent cytoreductive nephrectomy prior to administration of systemic immunotherapy. Furthermore, subgroup analysis of the Phase III clinical trial of sunitinib versus IF-α showed that patients who had prior cytoreductive nephrectomy had statistically longer PFS than patients who underwent IF-α alone with the primary tumor *in situ*.[70]

However, recommending cytoreductive nephrectomy in the era of molecular targeted therapy based on evidence extrapolated from trials conducted with immunotherapy is potentially problematic for the following reasons. Firstly, the premise that cytoreductive surgery may enhance subsequent immunotherapy by removing an immunosuppressive sink does not apply to molecular targeted therapy as the mechanism of action of these new class of drugs is mediated through the growth factor signalling pathway rather than immunologically mediated. Secondly, unlike immunotherapy, molecular targeted therapy has been shown to result in a primary tumor's response, which has rendered the rational to remove the primary lesion at the initial setting less compelling. Thus, without evidence from a well-designed Phase III trial comparing molecular targeted therapy alone versus a combination of cytoreductive surgery followed by molecular targeted therapy, the role of cytoreductive nephrectomy in the era of molecular targeted therapy has not been defined. Nevertheless, it may not be practical to conduct a clinical trial to assess the role of cytoreductive nephrectomy in the era of molecular targeted therapy especially in the setting of multiple drugs that need to be tested. Given the limitations, available data seems to suggest that, without evidence to the contrary, cytoreductive nephrectomy should be considered for those patients with good surgical risk harbouring a symptomatic primary tumor in the setting of limited metastatic burden.

The timing of initiating molecular targeted therapy in relation to cytoreductive surgery, when indicated, is currently being evaluated in clinical trials.[71,72] In the absence of available data, pre-nephrectomy systemic administration of molecular targeted therapy has the advantage of potential downstaging of the primary tumor. More importantly, until clinical and laboratory biomarkers to predict tumor response to molecular targeted therapy are available in the future, this approach can potentially allow selection of patients who will most likely benefit from cytoreductive surgery based on their response to the initial molecular targeted therapy.

### Surgical safety in the setting of molecular targeted therapy

Data addressing the safety of surgery in patients who had prior systemic treatment with molecular targeted therapy is scanty. Preclinical studies have shown possible complications with hemorrhage, thrombo-embolic events, and possible impaired wound healing with the use of bevacizumab and other small molecular inhibitors approved for used in patients with advanced RCC.[73,74] However, available data seems to suggest that patients undergoing surgery who had prior treatment with molecular targeted therapy do not seem to incur additional surgical risks in terms of blood loss, duration of anaesthesia, would healing, thrombo-embolic and cardiovascular-related complications, and duration of hospital stay.[75–77] More studies designed to assess the surgical safety issues related to the use of these new therapeutic agents are needed to better define the risk of surgery in this setting.

### The role of molecular targeted therapy in an adjuvant setting

There is a strong argument for adjuvant treatment with molecular targeted judging from the observation that up to one-third of patients may develop metastatic disease post-radical nephrectomy for localized disease. However, there is a paucity of evidence to support the use of molecular targeted therapy after nephrectomy for RCC at this stage. Currently, there are two large randomized Phase III trials being conducted in the United States and Europe to investigate the efficacy of these new agents in an adjuvant setting. The ECOG Intergroup Trial E2805 will investigate the efficacy of sunitinib and sorafenib as adjuvant treatment with a primary endpoint of disease-free survival. The European study- the SORCE trial– was organized by the Medical Research Council of UK and is currently accruing patients who are at high risk for metastatic recurrence after nephrectomy to either a one- or three-year duration of sorafenib. Currently, given that long-term administration of these agents may be required as adjuvant therapy, the potential toxicities associated with these drugs [Table 3] and the high cost involved, the use of molecular targeted therapy in an adjuvant setting is currently not recommended outside the context of a clinical trial.

**Table 3:** Reported adverse reactions associated with the three commonly used molecular targeted drugs in advanced RCC

	Sunitinib	Sorafenib	Temsirolimus
Common (≥20%)	Fatigue, asthenia, hypothyroidism, diarrhea, nausea, mucositis/stomatitis, vomiting, dyspepsia, abdominal pain, constipation, hypertension, rash, hand-foot syndrome, skin discoloration, altered taste, anorexia, and bleeding	Fatigue, weight loss, rash/desquamation, hand-foot skin reaction, alopecia, diarrhea, anorexia, nausea, abdominal pain, laboratory abnormalities: lymphopenia, anemia, neutropenia, hypophosphataemia, elevated lipase/amylase	Rash, asthenia, mucositis, nausea, edema, anorexia, impaired wound healing, laboratory abnormalities: anemia, hyperglycemia, hyperlipemia, hypertriglyceridemia, lymphopenia, elevated alkaline phosphatase/creatinine, hypophosphatemia, thrombocytopenia and leukopenia.
Uncommon and potentially serious adverse effects	Left ventricular dysfunction, QT interval prolongation, hemorrhage, hypertension, adrenal dysfunction	Hypertensive crisis, myocardial ischemia and/or infarction, congestive heart failure	Interstitial lung disease, thromboembolism

## WHAT IS NEW ON THE HORIZON?

### New molecular targets

There is clearly a need to identify new and more effective molecular targets to treat advanced RCC. New targets will also need to be tested in combination with currently available TKIs to overcome the potential limitations of monotherapy. In this regard, our strategy is to explore the up-stream molecular targets that are known to regulate TKRs that play an important role in the pathogenesis of RCC (i.e., VEGFR and PDGFR). We have identified IGF1R as a potential candidate. The IGF1R is a member of the TKR family, which also includes the insulin receptor (IR). The IGF1R gene is located on chromosome 15q26 and encodes a single polypeptide of 1367 amino acids that is constitutively expressed in almost every cell. Multiple lines of evidence implicate the IGF1R and its ligands in the development and progression of cancer.[78,79] Firstly, the IGF1R plays a critical role in the establishment and maintenance of cell transformation as measured by the ability to grow in anchorage-independent conditions and to form tumors in mice.[80,81] Secondly, the IGF1R is frequently overexpressed by human cancers, including cancers of the colon,[46,82–84] myeloma,[85] melanoma,[86] ovary,[87] and prostate.[88] Thirdly, IGF1R activation or overexpression mediates many aspects of the malignant phenotype. Importantly, in the context of developing new treatment for mRCC, IGF1R signalling has been shown to regulate HIF1-α (manuscript in press), which is a master regulator of hypoxia inducible genes including VEGFR, PDGFR, and TGF-α all of which play important roles in the development of CC-RCC.

### IGF1R and RCC

A series of studies from the Mayo Clinic suggest that IGF1R expression is of particular importance in RCC. Approximately 50% of RCCs show detectable immunohistochemical staining for the IGF1R and this positive staining correlates with a higher grade of tumor and with poor prognosis even in low stage disease.[89–92] We observed that IGF1R expression in CC-RCC is regulated by the VHL gene. Inactivating VHL mutations occur in approximately 75% of CC-RCC cases.[13] We elucidated that pVHL suppresses IGF1R expression in human CC-RCC cells at the transcriptional level by sequestration of the Sp1 transcription factor. In addition, the VHL tumor suppressor also regulates the stability of *IGF1R* mRNA by interacting with the HuR RNA binding protein.[36] This is subsequently proven to be a significant contributor to renal tumorigenesis and also to chemorefractory (manuscript in press). These data and the oncogenic property of the IGF1R suggest that IGF1R is an attractive target for treatment of advanced RCC.

## PREDICTORS OF TREATMENT RESPONSE

In contrast to Her2 (breast cancer) and EGF (lung cancer) receptor inhibitor therapies where receptor overexpression in the former and tyrosine kinase mutation status in the latter could be used to guide treatment, no such correlation has been identified so far that predicts sensitivity to these molecular targeted agents in RCC. Understandably, there is currently an urgent need to establish predictors of clinical response to molecular targeted therapeutics in these groups of patients. It may be possible to use genomic and proteomic techniques as has been the case for EGFR inhibitors[93] and RCC-derived xenograft model to identify molecular markers of response. Our study employed an approach using human RCC-derived mouse xenograft model for preclinical drug testing and molecular and genetic profiling to develop genetic and biomakers to predict treatment responses using this new class of therapeutics. This approach has its advantages as conducting similar trials clinically is costly, time-consuming, and may not be ethically appropriate. When available, clinical and laboratory predictors for treatment responses will allow clinicians to identify patients with mRCC who are likely to respond to the currently available molecular targeted agents thus allowing selection of patients likely to be sensitive to this approach and allowing the use of the lowest possible effective doses. This will avoid unnecessary costs and side effects associated with these new drugs. Research in this area is actively being pursued by our laboratory and the outcomes are eagerly anticipated in the near future.

In summary, the small molecule inhibitors including sunitinib, sorafenib and mTOR inhibitors (temsirolimus and everolimus), and the combination of bevacizumab and erlotinib have been shown to demonstrate anti-tumor activity against RCC. Superior activity has been observed with sunitinib and temsirolimus as first-line therapy compared with cytokine therapy. As second-line therapy in cytokine-refractory patients, sorafenib and bevacizumab have been found to improve PFS and everolimus demonstrated clinical efficacy in patients who developed resistance to VEGF-based TKIs. It is clear that molecular targeted therapies are rapidly changing the management of mRCC. However, the issues of treatment resistance, lack of biomarkers to predict treatment response, and the unresolved issues of the role of surgery in the era of molecular targeted therapy will need to be addressed through further research.
